# Tic40 is important for reinsertion of proteins from the chloroplast stroma into the inner membrane

**DOI:** 10.1111/j.1365-313X.2008.03638.x

**Published:** 2008-08-20

**Authors:** Chi-Chou Chiu, Hsou-min Li

**Affiliations:** Institute of Molecular Biology, Academia SinicaTaipei 11529, Taiwan

**Keywords:** Tic40, chloroplasts, post-import, inner membrane, membrane protein insertion, proline

## Abstract

Chloroplast inner-membrane proteins Tic40 and Tic110 are first imported from the cytosol into the chloroplast stroma, and subsequently reinserted from the stroma into the inner membrane. However, the mechanism of reinsertion remains unclear. Here we show that Tic40 itself is involved in this reinsertion process. When precursors of either Tic40 or a Tic110 C-terminal truncate, tpTic110-Tic110N, were imported into chloroplasts isolated from a *tic40*-null mutant, soluble Tic40 and Tic110N intermediates accumulated in the stroma of *tic40*-mutant chloroplasts, due to a slower rate of reinsertion. We further show that a larger quantity of soluble Tic21 intermediates also accumulated in the stroma of *tic40*-mutant chloroplasts. In contrast, inner-membrane insertion of the triose-phosphate/phosphate translocator was not affected by the *tic40* mutation. Our data suggest that multiple pathways exist for the insertion of chloroplast inner-membrane proteins.

## Introduction

Although the chloroplast has its own genome, most chloroplast proteins are encoded by the nuclear genome in a precursor form with a transit peptide located at the N-terminus. Import of the precursor proteins into chloroplasts is mediated by the protein translocon complex, composed of the Toc (translocon at the outer envelope membrane of chloroplasts) and Tic (translocon at the inner envelope membrane of chloroplasts) proteins and stromal chaperones (Inaba and Schnell, 2008; Soll and Schleiff, 2004).

Chloroplasts have a complicated structure with three membrane systems: the outer and inner membranes of the envelope, and the thylakoid membrane. Correct and efficient insertion of chloroplast membrane proteins into these membranes is essential for chloroplast biogenesis. There are many studies analyzing protein insertion into the outer envelope and the thylakoid membranes (Hofmann and Theg, 2005; Jarvis and Robinson, 2004; Tu *et al.*, 2004), but only a limited number of studies have addressed the molecular mechanism of insertion of protein into the inner envelope membrane.

The chloroplast inner envelope membrane contains many important proteins, including metabolite transporters, protein translocon components and enzymes for lipid biosynthesis. Targeting and insertion for only a few of these inner-membrane proteins have been studied. The transit peptide of phosphate translocator (PHT) has been shown to function as a stroma-targeting signal, with an inner-membrane insertion signal contained within the N-terminal hydrophobic region of the mature protein. No soluble targeting intermediate has been observed, suggesting that PHT probably directly diffuses laterally into the inner membrane from the inner-membrane protein-translocation channel (Brink *et al.*, 1995; Knight and Gray, 1995).

In contrast to PHT, Tic40 and Tic110 have been shown to follow a ‘post-import’ pathway, in which they are first imported into the stroma, producing soluble intermediates, which then insert into the inner membrane from the stroma (Li and Schnell, 2006; Lübeck *et al.*, 1997). Tic110, the major Tic protein identified (Kessler and Blobel, 1996; Lübeck *et al.*, 1996), most likely functions as the stroma-side receptor for transit peptides and the scaffold for translocation of precursors across the inner membrane (Inaba *et al.*, 2003, 2005). Tic40 is a co-chaperone that coordinates Tic110 and the stromal chaperone Hsp93 during protein translocation across the inner membrane (Chou *et al.*, 2006). The transit peptide of Tic40 is processed in two steps. The first part of the transit peptide is cleaved in the stroma to produce the stromal intermediate iTic40, which is intermediate in size between the precursor and the mature protein (Li and Schnell, 2006; Tripp *et al.*, 2007). The second part of the transit peptide is cleaved during or after the reinsertion. However, the signal for reinsertion is contained within the N-terminal serine/proline-rich region of the mature protein, not in the second part of the transit peptide (Li and Schnell, 2006; Tripp *et al.*, 2007). The transit peptide of Tic110 is processed only once, and therefore the stromal intermediate is the same size as the mature protein (Li and Schnell, 2006; Lübeck *et al.*, 1997). It is not clear how these stromal intermediates are inserted into the inner membrane. It has been shown that proteinaceous components of the inner membrane, but not those of the stroma, are required for the reinsertion, and that exogenous ATP and GTP are not required (Li and Schnell, 2006). However, another report has shown that ATP is required and has also suggested that stromal Hsp93 may be involved in the reinsertion of Tic110 (Vojta *et al.*, 2007).

Reinsertion of Tic110 from the stroma seems to be defective in transgenic Arabidopsis plants overexpressing a C-terminal fragment of Tic110 (Tic110C), because more stromal Tic110 accumulated in the Tic110C overexpressing plants (Inaba *et al.*, 2005). Recently, we have shown that Tic40 interacts with the C-terminal portion of Tic110 (Chou *et al.*, 2006). Therefore, it is possible that part of the effect of Tic110C overexpression may be due to high levels of Tic110C interfering with the function of Tic40. In this paper we investigate the possible involvement of Tic40 in inner-membrane-protein reinsertion by studying the reinsertion in *tic40*-null mutant chloroplasts. Our data suggest that Tic40 is important for the reinsertion of Tic40 and Tic110 from the stroma. In addition, in the light of additional analyses of the import of Tic21 and PHT, we suggest that multiple pathways exist for insertion of chloroplast inner-membrane proteins.

## Results

### Larger amounts of in vitro imported soluble iTic40 and Tic110N accumulated in tic40-mutant chloroplasts

To investigate if Tic40 was involved in the reinsertion of Tic40 and Tic110, [^35^S]-prTic40 and [^35^S]-tpTic110-Tic110N (identical to tp110-110N in Lübeck *et al.*, 1997) were imported into chloroplasts isolated from *tic40-1*, a *tic40*-null mutant (Chou *et al.*, 2003) and from wild-type seedlings. The import of precursors of the stromal RuBP carboxylase small subunit (prRBCS) and the thylakoid chlorophyll *a*/*b* binding protein (prCAB) served as controls. tpTic110-Tic110N is a pea Tic110 precursor truncate consisting of only the first 269 residues of the 997-residue full-length precursor. tpTic110-Tic110N contains all the information required for correct targeting and insertion into the inner membrane (Lübeck *et al.*, 1997). Because of its smaller size its import efficiency is higher, and the two forms (before and after removal of the transit peptide) are easier to distinguish on gels.

**Figure 1:**
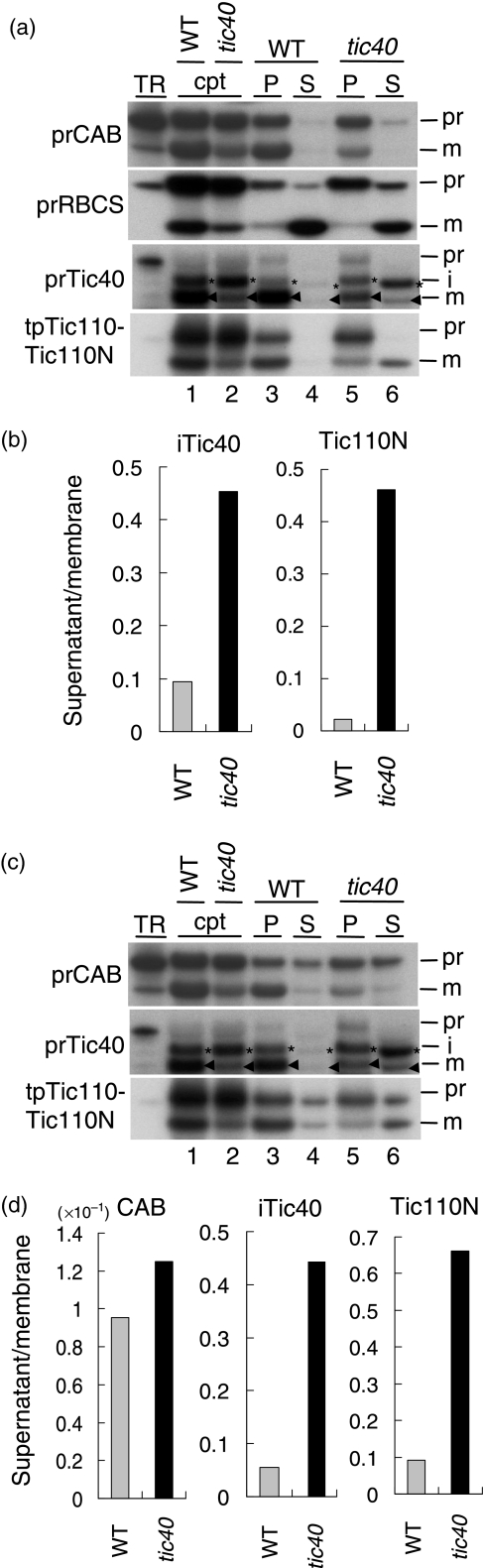
More *in vitro* imported iTic40 and Tic110N accumulated in the soluble fraction of *tic40*-mutant chloroplasts. (a, c) [^35^S]-prCAB, prRBCS, prTic40 and tpTic110-Tic110N were imported into wild-type (WT) and *tic40*-null mutant chloroplasts for 20 min. Re-isolated intact chloroplasts (cpt) were separated into pellet (P) and supernatant (S) fractions by hypotonic lysis in 0.2 m NaCl (a) or by alkaline extraction (c). Samples were analyzed by SDS-PAGE and fluorography. TR, 2.5% of *in-vitro* translated precursor proteins added to the import reactions. pr, precursor form; i, intermediate; m, mature form. iTic40 and mature Tic40 are indicated with an asterisk and an arrowhead, respectively. (b, d) Ratio of soluble to membrane-inserted proteins in (a) and (c), respectively. In the gels shown in (a) and (c), an equal amount of proteins from the WT and *tic40*-mutnat pellets were loaded (lanes 3 and 5). Because *tic40*-mutant chloroplasts contain less protein (Chou *et al.*, 2003), slightly more chloroplasts were loaded from the mutant. Each supernatant fraction then used three times the number of chloroplasts of its corresponding pellet fraction in order to have similar band intensities across the gel. The quantification in (b) and (d) has been corrected for the chloroplast numbers and represents the ratio within a chloroplast.

As shown in Figure 1, less mature CAB and RBCS were imported into *tic40*-mutant chloroplasts (Figure 1a, lane 2), confirming the general nature of the *tic40*-mutant chloroplast defect in protein import (Chou *et al.*, 2003). When chloroplasts were hypotonically lysed in 0.2 m NaCl (Li and Schnell, 2006) and separated into soluble and membrane fractions by centrifugation, mature CAB was found exclusively in the membrane fraction and most of the mature RBCS was in the soluble fraction (Figure 1a).

In contrast, although less mature Tic40 and Tic110N were imported into the *tic40*-mutant chloroplasts (Figure 1a, lane 2), when chloroplasts were lysed in 0.2 m NaCl, a much larger amount of iTic40 and Tic110N was detected in the soluble fraction of *tic40*-mutant chloroplasts (Figure 1a, lane 6). The ratio of soluble to membrane-inserted iTic40 and Tic110N was greatly increased in *tic40*-mutant chloroplasts (Figure 1b). This result suggested that the *tic40* mutation resulted in the accumulation of soluble iTic40 and Tic110N. An increased amount of mature Tic40 was also observed in the soluble fraction of *tic40*-mutant chloroplasts. Since iTic40 is thought to be the pathway intermediate for Tic40 reinsertion (Li and Schnell, 2006; Tripp *et al.*, 2007), only iTic40 was quantified.

Alkaline extraction is a more stringent test of membrane insertion than high-salt wash, and has also been used to separate the soluble intermediate from the membrane-inserted Tic40 (Tripp *et al.*, 2007). To reveal the true extent of reduction of iTic40 and Tic110N insertion by *tic40*, we performed the same import experiments but separated the membrane from the soluble fractions by alkaline extraction. As shown in Figure 1(c), a small amount of mature CAB was released to the soluble fraction by alkaline extraction and could therefore be quantified. The ratio of soluble to membrane-bound CAB was similar in the mutant and in the wild type (Figure 1d), indicating that *tic40* mutation did not affect insertion of CAB into the thylakoid. In comparison, a much greater amount of soluble iTic40 and Tic110N was again observed in the *tic40*-mutant chloroplasts. The ratio of soluble to membrane-inserted iTic40 and Tic110N was greatly increased in *tic40*-mutant chloroplasts (Figure 1d).

### In vitro imported iTic40 and Tic110N accumulated in the stroma of tic40-mutant chloroplasts

**Figure 2:**
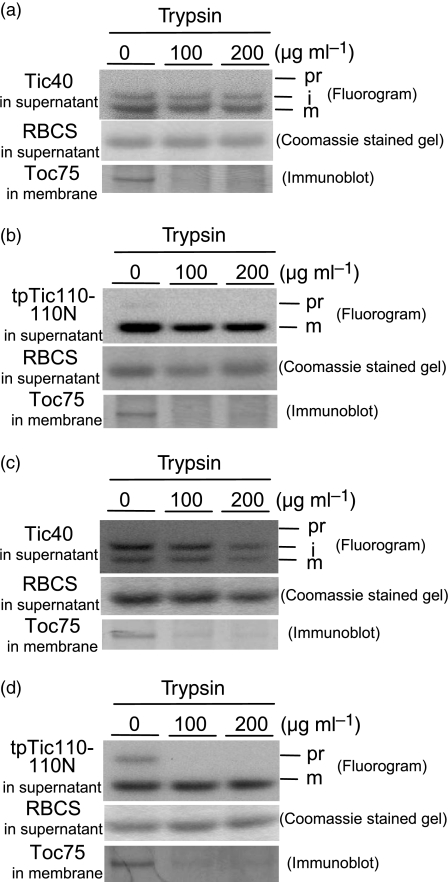
*In vitro* imported iTic40 and Tic110N accumulated in the stroma of *tic4*0-mutant chloroplasts. [^35^S]-prTic40 and [^35^S]-tpTic110-Tic110N were imported into *tic40*-mutant chloroplasts for 20 min. After import chloroplasts were treated with various concentrations of trypsin. Re-isolated intact chloroplasts were separated into pellet (for analysis of Toc75) and supernatant [for analysis of Tic40, Tic110N and RuBP carboxylase small subunit (RBCS)] fractions by hypotonic lysis in 0.2 m NaCl (a, b) or by alkaline extraction (c, d). Samples were analyzed by SDS-PAGE. Proteins were detected by fluorography (Tic40 and Tic110N), Coomassie blue staining (RBCS) or immunoblots (Toc75).

We next investigated if the soluble iTic40 and Tic110N detected in *tic40*-mutant chloroplasts were localized in the stroma, as observed in wild-type chloroplasts. Chloroplasts were re-isolated after import of [^35^S]-prTic40 and [^35^S]-tpTic110-Tic110N, treated with an increasing concentration of trypsin, lysed in 0.2 m NaCl and the soluble fraction was then collected by centrifugation. As trypsin can only penetrate the outer membrane and not the inner membrane (Jackson *et al.*, 1998), we were able to distinguish whether the soluble iTic40 and Tic110N were outside the inner membrane or in the stroma. As a control, Toc75, an integral outer-membrane protein from the membrane fraction, was also analyzed. As shown in Figure 2(a,b), Toc75 was nearly completely degraded by the trypsin treatment. In contrast, both iTic40 and Tic110N in the soluble fraction were resistant to trypsin digestion, indicating that they were localized in the stroma of *tic40*-mutant chloroplasts. Similar results were obtained when the soluble fraction was collected by alkaline extraction and centrifugation (Figure 2c,d), indicating that soluble iTic40 and Tic110N released by alkaline extraction were also located in the stroma.

### Stromal iTic40 inserted into the inner membrane with a slower rate in tic40-mutant chloroplasts

**Figure 3:**
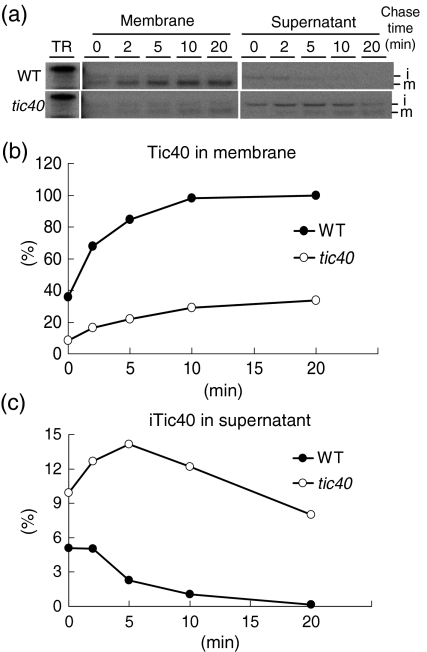
Accumulation kinetics of membrane-inserted Tic40 and soluble iTic40 in wild-type (WT) and *tic40*-mutant chloroplasts. (a) [^35^S]-prTic40 was pre-imported into WT and *tic40*-mutant chloroplasts for 2 min, re-isolated and then chased for the length of time indicated. Chloroplasts were treated with thermolysin and separated into membrane and supernatant fractions by alkaline extraction. Samples were analyzed by SDS-PAGE and fluorography. An equal amount of protein from all membrane-fraction samples was loaded, and each supernatant fraction then used three times the number of chloroplasts of its corresponding membrane fraction. TR, *in vitro* translated protein. i, intermediate; m, mature form. All WT samples were analyzed on one gel and all mutant samples on another. All gels were exposed for the same amount of time, except that the TR-lane image was from gels exposed for 1/6 the amount of time. (b) Quantification of the membrane-inserted mature Tic40 in (a). (c) Quantification of iTic40 in the supernatant fractions of (a). For both (b) and (c), the amount of membrane-inserted mature Tic40 in WT chloroplasts at 20 min was set as 100%.

Accumulation of iTic40 and Tic110 in the stroma of *tic40*-mutant chloroplasts could be a result of a slower insertion rate of these intermediates. It is also possible that, in the absence of Tic40, some intermediates could not be properly transferred to the reinsertion machinery and thus accumulated in the stroma. To investigate these possibilities, we tested whether the iTic40 and Tic110 that accumulated in the stroma of *tic40*-mutant chloroplasts were still competent in inserting into the inner membrane. We analyzed the accumulation kinetics of stromal iTic40 in wild-type and *tic40*-mutant chloroplasts using a two-step import reaction consisting of a pre-import step and a chase step. In the pre-import step, [^35^S]-prTic40 was incubated with isolated chloroplasts for 2 min under normal import conditions with 3 mm ATP. Chloroplasts at this stage should contain active early import intermediates of various stages *en route* to their final destination. Due to the slower import rate and low import efficiency of Arabidopsis chloroplasts compared with pea chloroplasts, we found that this condition was more effective in accumulating greater amounts of active import intermediates than arresting import at the binding stage using low (<100 μm) ATP (data not shown). After the 2-min pre-import, chloroplasts were washed and resuspended in import buffer with 3 mm ATP to initiate the chase. At each time point during the chase, a proportion of the chloroplasts were withdrawn and treated with thermolysin to remove surface-bound non-imported molecules. Intact chloroplasts were re-isolated and separated into membrane and supernatant fractions by alkaline extraction (Figure 3a). Alkaline extraction was used because it is better at revealing truly membrane-inserted protein, and it released a greater quantity of soluble intermediates which made detection easier. To facilitate direct comparison, the amount of membrane-inserted mature Tic40 at the end of the 20-min chase in the wild type was set as 100%. In wild-type chloroplasts, the amount of stromal iTic40 was only about 5% of this level at the beginning of the chase and decreased rapidly thereafter (Figure 3c, filled circles). On the other hand, the membrane-inserted Tic40 increased from 36% to 98% during the first 10 min (Figure 3b, filled circles). These results indicated that reinsertion under normal conditions was an extremely rapid process, and in most experiments, stromal intermediate could not be observed by our method of stopping the import.

In comparison, in *tic40*-mutant chloroplasts the amount of stromal iTic40 actually increased over the first 5 min of the chase and reached ∼14% (Figure 3c, open circles). It then gradually decreased in line with the rise in membrane-inserted mature Tic40 (Figure 3b, open circles). These results suggested that the stromal iTic40 accumulated in *tic40* chloroplasts was a pathway intermediate *en route* for insertion into the inner membrane. The slower insertion rate in the *tic40* mutant facilitated the observation of this intermediate step. A small amount of iTic40 remained in the stroma even after a 40-min chase (data not shown), suggesting that in the absence of Tic40, some stromal iTic40 eventually lost competence for inserted. We also tried to remove surface-bound precursors by thermolysin treatment before the chase, leaving only the stromal iTic40, in order to observe direct conversion of iTic40 into the membrane-inserted mature Tic40. However, due to the fragile nature of the *tic40*-mutant chloroplasts, the signals were too weak to be reliable.

### Stromal Tic110N inserted into the inner membrane at a slower rate in tic40-mutant chloroplasts

**Figure 4:**
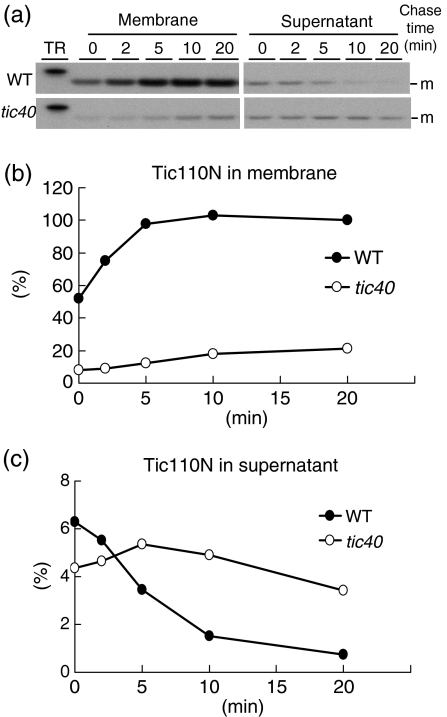
Accumulation kinetics of Tic110N in the membrane and supernatant fractions in wild-type (WT) and *tic40*-mutant chloroplasts. (a) [^35^S]-tpTic110-Tic110N was pre-imported into WT and *tic40*-mutant chloroplasts for 2 min, re-isolated and then chased for the length of time indicated. Chloroplasts were treated with thermolysin and separated into pellet and supernatant fractions by alkaline extraction. Samples were analyzed by SDS-PAGE and fluorography. An equal amount of protein from all membrane-fraction samples was loaded, and each supernatant fraction then used three times the number of chloroplasts of its corresponding membrane fraction. TR, *in-vitro* translated protein. m, mature form. All wild-type samples were analyzed on one gel and all mutant samples on another. All gels were exposed for the same amount of time. (b) Quantification of the membrane-inserted mature Tic110N in (a). (c) Quantification of Tic110N in the supernatant fractions of (a). For both (b) and (c), the amount of membrane-inserted matureTic110N in the wild type at 20 min was set as 100%.

We then performed the same pre-import-and-chase experiment with [^35^S]-tpTic110-Tic110N. In wild-type chloroplasts, the amount of stromal Tic110N rapidly decreased during the chase (Figure 4c, filled circles), similar to what was observed for iTic40. In contrast, in *tic40*-mutant chloroplasts, the amount of stromal Tic110N first rose then decreased (Figure 4c, open circles), suggesting that in the absence of Tic40, the reinsertion of stromal Tic110N was slower, which allowed transient accumulation of stromal Tic110N.

### Stromal iTic21 inserted into the inner membrane at a slower rate in tic40-mutant chloroplasts

**Figure 5:**
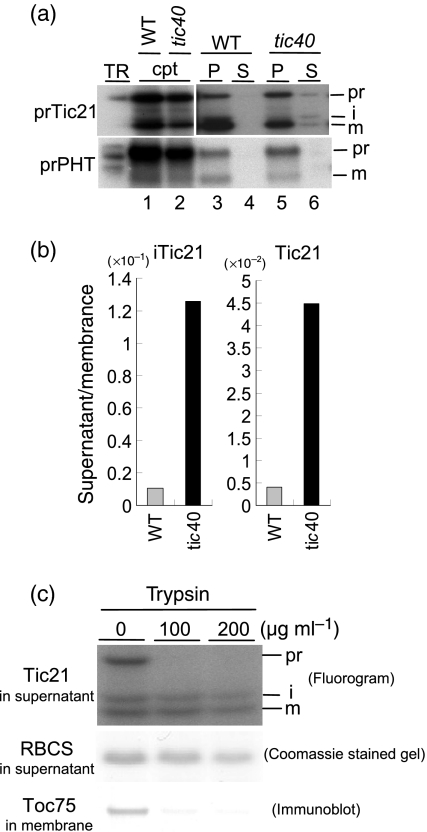
Tic40 is important for the membrane insertion of Tic21 but not phosphate translocator (PHT). (a) [^35^S]-prTic21 and [^35^S]-prPHT were imported into wild-type (WT) and *tic40*-mutant chloroplasts for 20 min. Re-isolated intact chloroplasts (cpt) were separated into pellet (P) and supernatant (S) fractions by hypotonic lysis in 0.2 m NaCl. Samples were analyzed by SDS-PAGE and fluorography. TR, 2.5% of *in vitro* translated precursor proteins added to the import reactions. pr, precursor form; i, intermediate; m, mature form. For the prTic21 panel, the TR and cpt portion was a shorter exposure from the same gel of the pellet (P) and supernatant (S) portion. (b) Ratio of supernatant to membrane-inserted iTic21 and mature Tic21 as shown in (a). In the gels shown in (a), an equal amount of protein from the WT and *tic40*-mutant pellets was loaded (lanes 3 and 5). Each supernatant fraction then used three times the number of chloroplasts of its corresponding pellet fraction in order to have similar band intensities across the gel. The quantification in (b) has been corrected for the chloroplast numbers and represents the ratio within a chloroplast. (c) *In vitro* imported iTic21 and mature Tic21 accumulated in the stroma of *tic40*-mutant chloroplasts. [^35^S]-prTic21 was imported into *tic40*-mutant chloroplasts for 20 min. After import chloroplasts were treated with various concentrations of trypsin. Re-isolated intact chloroplasts were separated into pellet (for analyzing Toc75) and supernatant [for analyzing Tic21and RuBP carboxylase small subunit (RBCS)] fractions by hypotonic lysis in 0.2 m NaCl. Samples were analyzed by SDS-PAGE. Proteins were detected by fluorography (Tic21), Coomassie blue staining (RBCS) or immunoblots (Toc75).

To investigate if Tic40 is also important for the insertion of other inner-membrane proteins, we examined the import of two other [^35^S]-labeled Arabidopsis inner-membrane precursor proteins, prTic21 (Teng *et al.*, 2006) and prPHT (triose-phosphate/phosphate translocator) (Schneider *et al.*, 2002) (Figure 5a). Chloroplasts after import were separated into membrane and supernatant fractions by hypotonic lysis in 0.2 m NaCl and centrifugation.

Processing of the prTic21 transit peptide most likely occurs in two steps since an intermediate-sized protein slightly larger than the mature protein, hereafter referred as iTic21, can be observed (Duy *et al.*, 2007; Teng *et al.*, 2006). Interestingly, as observed for iTic40 and Tic110N, while most of the iTic21 in wild-type chloroplasts was located in the membrane fraction (Figure 5a, lane 3), most iTic21 in *tic40*-mutant chloroplasts was located in the soluble fraction (lane 6). A substantial amount of mature Tic21 was also located in the soluble fraction of *tic40*-mutant chloroplasts. In comparison, no soluble PHT was found either in the mutant or wild-type chloroplasts, agreeing with previous observations that inner-membrane insertion of PHT probably did not proceed through a soluble intermediate (Brink *et al.*, 1995; Knight and Gray, 1995).

To determine the location of the soluble iTic21 and Tic21 observed in *tic40*-mutant chloroplasts, chloroplasts were re-isolated after import of [^35^S]-prTic21, treated with an increasing concentration of trypsin and lysed in 0.2 m NaCl. The soluble fraction was collected by centrifugation. Toc75 was again used as a control for the effectiveness of the trypsin treatment. As shown in Figure 5(c), Toc75 was nearly completely degraded by the trypsin treatment. In contrast, iTic21 and Tic21 in the supernatant fraction were resistant to trypsin digestion, indicating that they were localized in the stroma of *tic40*-mutant chloroplasts.

**Figure 6:**
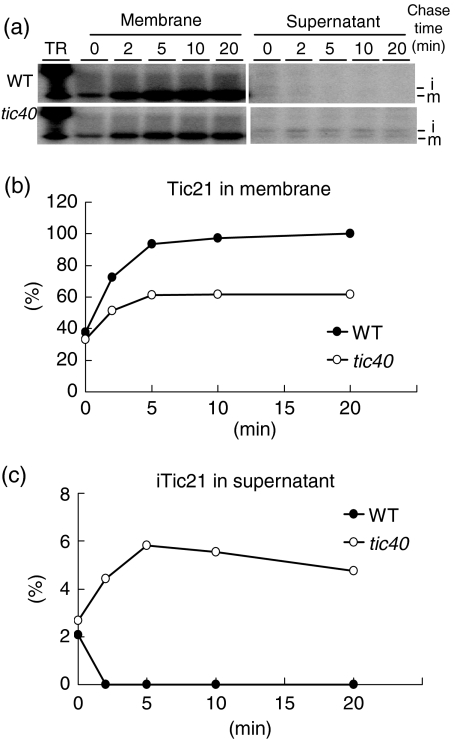
Accumulation kinetics of membrane-inserted Tic21 and soluble iTic21 in wild-type (WT) and *tic40*-mutant chloroplasts. (a) [^35^S]-prTic21 was pre-imported into WT and *tic40*-mutant chloroplasts for 2 min, re-isolated and then chased for the length of time indicated. Chloroplasts were treated with thermolysin and separated into membrane and supernatant fractions by alkaline extraction. Samples were analyzed by SDS-PAGE and fluorography. An equal amount of protein from all membrane-fraction samples was loaded, and each supernatant fraction then used three times the number of chloroplasts of its corresponding membrane fraction. TR, *in vitro* translated protein. i, intermediate; m, mature form. All WT samples were analyzed on one gel and all mutant samples on another. All gels were exposed for the same amount of time. (b) Quantification of the membrane-inserted mature Tic21 in (a). (c) Quantification of iTic21 in the supernatant fractions of (a). For both (b) and (c), the amount of membrane-inserted mature Tic21 in WT chloroplasts at 20 min was set as 100%.

We next analyzed the accumulation kinetics of stromal iTic21 using the same pre-import-and-chase experiment. In wild-type chloroplasts, a very small amount of stromal iTic21 was detected only at the beginning of the chase (Figure 6c, filled circles). In contrast, in *tic40*-mutant chloroplasts the amount of stromal iTic21 was higher (Figure 6c, open circles) and it first increased and then decreased, similar to what was observed for soluble iTic40 and Tic110N (Figures 3c and 4c).

## Discussion

In this study, we found that Tic40 is important for reinsertion of Tic40, Tic110 and Tic21 from the stroma into the inner membrane. A larger quantity of soluble intermediates of these proteins was detected during *in vitro* import assays using isolated *tic40*-mutant chloroplasts. In comparison, PHT appeared to insert into the inner membrane through a Tic40-independent pathway. No soluble PHT was detected, which is consistent with a previous suggestion that PHT inserts into the inner membrane through a stop-transfer pathway (Brink *et al.*, 1995; Knight and Gray, 1995; Li *et al.*, 1992).

In mitochondria, inner-membrane protein insertion has been shown to occur through at least three pathways (Neupert and Herrmann, 2007). First, carrier proteins with internal targeting signals insert into the inner membrane through the Tim22 complex by lateral diffusion. Second, some pre-sequence-containing inner-membrane proteins diffuse laterally into the inner membrane through the Tim23 complex. Third, other pre-sequence-containing inner-membrane proteins go through the conservative sorting, or post-import, pathway. They are first fully translocated into the matrix through the Tim23 complex, and then reinsert into the inner membrane from the matrix. Proline residues in the transmembrane domain seem to be the determinant for pathway selection (Meier *et al.*, 2005). Proline residues are present in the transmembrane domains of most inner-membrane proteins that go through the conservative sorting pathway, but are almost entirely absent in transmembrane domains of proteins that go through the stop-transfer pathway. Accordingly, insertion or deletion of proline residues causes switching of the sorting pathway (Meier *et al.*, 2005).

**Figure 7:**
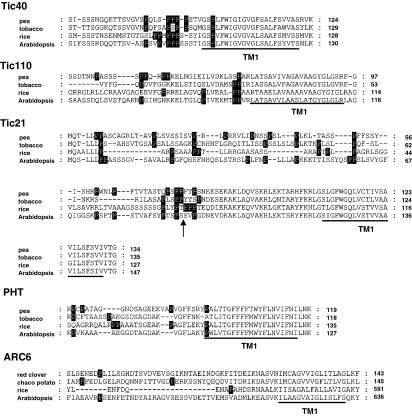
Conserved proline residues are present in Tic40, Tic110 and iTic21 but not in phosphate translocator (PHT) and accumulation and replication of chloroplasts 6 (ARC6). Sequence alignment of the N-terminal portion of prTic21 and mature Tic40, Tic110 and PHT, and the C-terminal portion of ARC6. Multiple sequence alignments were created using the Wisconsin Package (version 10.3). The transit-peptide processing site of Arabidopsis prTic21 is indicated with an upward arrow. First transmembrane domains (TM1) are underlined. Proline residues are highlighted in white letters on a black background. Residues are numbered with the initiation methionine as residue 1.

A serine/proline-rich domain located at the N-terminal portion, in front of the transmembrane domain of mature Arabidopsis Tic40, has been shown to be required for stromal reinsertion of Tic40 (Tripp *et al.*, 2007). A serine/proline-rich-like domain was also observed in the same region of pea Tic110 (Tripp *et al.*, 2007). We therefore analyzed the region in front of the first transmembrane domain of Tic40, Tic110, Tic21 and PHT from four representative species (pea or red clover, Arabidopsis, rice and one plant from the Solanaceae family). Because Tic21 seems to insert as the intermediate form iTic21, but the cleavage site that produces iTic21 is still not known, the transit peptide of prTic21 was also included. Accumulation and replication of chloroplasts 6 (ARC6), which has been suggested to use a stop-transfer pathway (Tripp *et al.*, 2007), was also analyzed. This protein is a tail-anchored protein, so the region around the transmembrane domain at the C-terminus of the protein was analyzed. As shown in Figure 7, conserved proline residues in front of the first transmembrane domain are present in the Tic40 and Tic110 analyzed but are absent in PHT and ARC6. Serines are usually, but not always, present in the vicinity of these conserved proline residues. Because deletion of a segment containing these proline residues in Arabidopsis Ti40 resulted in accumulation of Tic40 in the soluble fraction (Tripp *et al.*, 2007), we hypothesize that these proline residues may be important for recognition by the sorting/reinsertion machinery in the stroma. The conserved proline residues in iTic21 do not immediately precede the transmembrane domain but are found around the transit-peptide processing site. This may be the reason why the transit peptide of prTic21 is initially only partly processed – so that the conserved proline residues in iTic21 are available for recognition by the sorting/reinsertion machinery. Further experiments are required to verify the importance of these proline residues in inner-membrane reinsertion.

The Tic40 protein is the first inner-membrane component identified that assists in the stromal reinsertion of the post-import pathway. Two previous studies have investigated possible components required for reinsertion. Using inside-out inner-membrane vesicles, it has been shown that proteinaceous components at the inner membrane, but not stromal proteins or exogenous nucleoside triphosphate, are required for the reinsertion of Tic40 (Li and Schnell, 2006). However, another report has suggested that ATP hydrolysis and the stromal chaperone Hsp93 are required for Tic110 reinsertion (Vojta *et al.*, 2007). It is possible that Tic40 and Tic110 have different energy requirements for their reinsertion. It would be interesting to analyze whether more soluble Tic110 and iTic40 accumulate in the stroma of *hsp93*-mutant chloroplasts.

It is most likely that Tic40 plays an accessory role to other central components of the reinsertion machinery since its absence slows down but does not block the reinsertion. Tic40 has been shown to function as a co-chaperone that coordinates Tic110 and Hsp93 during translocation of precursor across the inner membrane (Bedard *et al.*, 2007; Chou *et al.*, 2003, 2006). In this study, we showed that Tic40 is also involved in the reinsertion of inner-membrane proteins from the stroma. It is possible that Tic40 is a member of two different complexes, the Tic complex and an unidentified reinsertion complex. If a separate complex for reinsertion does exist, results from previous studies suggest that Hsp93 and Tic110 are also likely to be components of such a complex (Li and Schnell, 2006; Vojta *et al.*, 2007). It is also possible that the complex formed by Tic40/Tic110/Hsp93 plays a dual role: in the general translocation of precursor proteins into the stroma, and in the reinsertion of inner-membrane proteins from the stroma. Furthermore, it is likely that Tic110 plays a more central role than Tic40, since endogenous Tic110 accumulated in the stroma of Tic110C-overexpressing plants (Inaba *et al.*, 2005) suggesting that insertion of some Tic110 was fully blocked. In the *tic40* mutant we could only detect accumulation of soluble intermediates from newly *in vitro* imported proteins but not a consistent accumulation of endogenous Tic110 or Tic40 (data not shown). This suggests that, although at a slower rate, most imported Tic110, Tic40 and Tic21 was eventually inserted into the inner membrane. Further experiments are required to elucidate the molecular mechanism of inner-membrane protein reinsertion.

## Experimental procedures

### Plant materials, growth conditions and chloroplast isolation

Arabidopsis plants (Ws ecotype) were grown on MS synthetic agar medium (Murashige and Skoog, 1962) with 2% sucrose as described previously (Chou *et al.*, 2003), except that plants were grown for 21–24 days. Isolation of chloroplasts from the wild type and the *tic40-1* mutant was performed as described (Perry *et al.*, 1991), except that the grinding buffer was modified to 300 mm sorbitol, 0.5% BSA, 50 mm HEPES-KOH, 2 mm EDTA, pH 8.0.

### Translation of precursor proteins

The full-length cDNA of Arabidopsis prTic40 (At5g16620) and prPHT (At5g46110) was amplified by RT-PCR using the following primers: for prTic40, *Sph*I-Tic40-F (5′-CCCGCATGCATGGAGAACCTTACCCTAGTTTCTT-3′) and *Kpn*I-Tic40-R (5′-CCCGGTACCTCAACCCGTCATTCCTGGGAAGAGCT-3′); and for prPHT, *Xba*I-atPHT-F (5′-CCCTCTAGAATGGAGTCACGCGTGCTGTTAC-3′) and *Kpn*I-atPHT-R (5′-CCCGGTACCCTATGCTTTCTTTCCTTGCCGT-3′). The amplified fragments of prTic40 and prPHT were subcloned into the *Sph*I/*Kpn*I site and the *Xba*I/*Kpn*I site of the pSP72 plasmid (Promega, http://www.promega.com/), respectively. The RNA was transcribed *in vitro* using T7 RNA polymerase (Promega) from plasmid containing cDNA for tpTic110-Tic110N or SP6 RNA polymerase (Promega) for prRBCS, prCAB, prTic40, prTic21 and prPHT. All precursor proteins were synthesized by *in vitro* translation with wheat germ extracts (Promega) programmed with *in vitro* transcribed RNA, except that reticulocyte lysates (Promega) was used for tpTic110-Tic110N.

### Protein import into chloroplasts and post-import treatments

Isolated chloroplasts from both *tic40-1* mutant and the wild type were adjusted to 1 mg chlorophyll ml^−1^ in import buffer (300 mm sorbitol, 50 mm HEPES-KOH, pH 8.0) and the same volume of mutant and wild-type chloroplasts was used for each experiment. For regular import, [^35^S]-labeled precursor proteins were incubated with isolated chloroplasts in the presence of 3 mm ATP in import buffer at room temperature for 20 min. The import reaction was stopped by adding an excess amount of ice-cold import buffer. Chloroplasts were pelleted, washed and then lysed with 0.2 m NaCl as described (Li and Schnell, 2006) except the HS buffer was changed to the import buffer, or alkaline-extracted with 0.1 m Na_2_CO_3_ (pH 11.5) for 30 min at 4°C. For pre-import (Figures 3, 4 and 6), [^35^S]-labeled precursor proteins were incubated with isolated chloroplasts in the presence of 3 mm ATP in import buffer at room temperature for 2 min. Pre-import was stopped by adding an excess amount of ice-cold import buffer. Chloroplasts were pelleted and resuspended in import buffer containing 3 mm ATP and chased for the amount of time specified. After the chase chloroplasts were treated with 100 μg ml^−1^ thermolysin (Fitzpatrick and Keegstra, 2001), re-isolated through a 35% Percoll cushion, and then alkaline-extracted with 0.1 m Na_2_CO_3_ (pH 11.5) for 30 min at 4°C. High-salt-lysed and alkaline-extracted samples were then separated into pellet and supernatant fractions by ultracentrifugation at 100 000 ***g*** for 45 min. Supernatant fractions were precipitated in 10% trichloroacetic acid, washed with 100% ice-cold acetone and dissolved in SDS-PAGE sample buffer. Trypsin treatment of chloroplasts after regular import was performed as described (Jackson *et al.*, 1998).
